# Characterizing Multistationarity Regimes in Biochemical Reaction Networks

**DOI:** 10.1371/journal.pone.0039194

**Published:** 2012-07-03

**Authors:** Irene Otero-Muras, Julio R. Banga, Antonio A. Alonso

**Affiliations:** 1 Department of Biosystems Science and Engineering, The Swiss Federal Institute of Technology Zurich, Zurich, Switzerland; 2 BioProcess Engineering Group, Instituto Investigaciones Marinas- Consejo Superior de Investigaciones Científicas, Spanish National Research Council, Vigo, Spain; Centre for Genomic Regulation (CRG), Universitat Pompeu Fabra, Spain

## Abstract

Switch like responses appear as common strategies in the regulation of cellular systems. Here we present a method to characterize bistable regimes in biochemical reaction networks that can be of use to both *direct* and *reverse* engineering of biological switches. In the design of a synthetic biological switch, it is important to study the capability for bistability of the underlying biochemical network structure. Chemical Reaction Network Theory (CRNT) may help at this level to decide whether a given network has the capacity for multiple positive equilibria, based on their structural properties. However, in order to build a working switch, we also need to ensure that the bistability property is robust, by studying the conditions leading to the existence of two different steady states. In the reverse engineering of biological switches, knowledge collected about the bistable regimes of the underlying potential model structures can contribute at the model identification stage to a drastic reduction of the feasible region in the parameter space of search. In this work, we make use and extend previous results of the CRNT, aiming not only to discriminate whether a biochemical reaction network can exhibit multiple steady states, but also to determine the regions within the whole space of parameters capable of producing multistationarity. To that purpose we present and justify a condition on the parameters of biochemical networks for the appearance of multistationarity, and propose an efficient and reliable computational method to check its satisfaction through the parameter space.

## Introduction

Multistability is a nonlinear phenomenon characterized by the existence of two or more stable steady states, where a given dynamical system will evolve depending on its initial conditions. Important biological phenomena, like cellular decision processes, rely on multistable models, where the different functional phenotypic states or cell fates can be understood as *discrete, stable and mutually exclusive* stable states [1]. Experimental evidences for bistability have been found in numerous pathways involved in cell decision processes, such as the p42 MAPK/Cdc2 network governing the maturation of oocytes in *Xenopus* [2], the pheromone sensing MAPK pathway in *S. cerevisiae* [3], or the Rb-E2F pathway regulating proliferation in mammalian cells [4]. The analysis of mathematical models of the underlying multistable networks contributes to understand these biological phenomena from a systems perspective. In [5] for example, basic design principles for the control of the cell cycle are suggested based on the modeling and analysis of the gene circuit underlying the Rb-E2F switch, identified by the criterion of robustness.

The dynamics of biochemical reaction networks (i.e., the time evolution of the vector of species concentrations) can be described by models of coupled ordinary differential equations where the structure depends on the reaction connectivities, stoichiometry and kinetics, and the parameters are defined from kinetic rate constants. Modeling a biochemical system consists of inferring the structure and parameters from experimental data. For a given model structure, the corresponding parameters are typically estimated from experimental time course measurements of observables (usually linear combinations of some subsets of the species concentrations), by minimizing some measure of error between the experimental data and the model prediction [6]. Nontraditional methodologies for the determination of reaction mechanisms from kinetic data sets have been reviewed by [7], [8]. In processes occurring within living cells, the access to quantitative information is often very limited, and this fact has severe implications on the development of mathematical models: it hampers model discrimination, often leads to poor parameter identifiability, and makes the parameter estimation task very challenging, since it entails to solve a nonconvex optimization problem in high dimensional search spaces [9], which cannot be reduced and/or constrained in absence of a priori knowledge about feasible parameter values.

One of the challenges of systems biology is to provide tools to overcome the lack of quantitative information, by exploring and exploiting connections between model structure and/or parameters with the expected dynamic behaviour [10]–[12]. In this context, methods to systematically detect multistationary regimes in biochemical systems will help modeling multistable systems, constraining the feasible parameter regions, for a given structure, based on the capability to produce multistationarity. These methods are also of great interest in the design of synthetic biological switches, where the robustness of the multistationarity property needs to be analyzed [13]–[15].

Current results in this direction are derived from different fields, from classical bifurcation theory [16] to monotone systems [17]. In particular, structural properties of reaction networks and their connection with multistationarity are at the core of the Chemical Reaction Network Theory, pioneered by Feinberg, Jackson and Horn [18], [19] and subject to ongoing development [20], [21] with special interest in the application to biological systems [22]–[24]. In the context of cell signaling, for example, CRNT has been used to discard kinetic mechanisms based on their capacity for multistationarity [25], [26].

The deficiency one algorithm, the advanced deficiency theory, the deficiency zero and deficiency one theorems are part of the CRNT in which networks are classified by means of a nonnegative integer index called *deficiency* –a property of the graph of complexes of a network– and some structural conditions are evaluated to decide whether networks have the capacity for multiple positive equilibria [27].

As pointed out in [28], CRNT provides surprisingly strong results for reaction networks based only on the systems structure. For example, the deficiency zero theorem asserts that every weakly reversible network of zero deficiency has a unique equilibrium, for any choices of parameter values. However, when multistationarity cannot be ruled out, nothing can be said about how the parameters affect the qualitative behaviour of the solutions.

In a previous paper [29] we have introduced the parameters into the picture, providing a canonical expression for the equilibrium manifold in terms of the kinetic parameters and the so called deficiency parameters of the network. The concept of network *layout*, introduced in [29] as the difference between the deficiency of a network (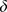) and the dimension of the equilibrium manifold (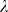), allowed us to classify biochemical reaction networks in three groups: *proper* networks (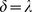), *overdimensioned* networks (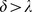) and *underdimensioned* networks (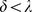). The analysis presented focused on proper networks, i.e. those networks where deficiency and manifold dimension coincide. For those networks, the qualitative behaviour of the manifold was evaluated through its derivative with respect to the deficiency parameters. Then a geometric intuitive idea was applied to find, under these restrictive assumptions (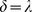), a condition on the parameters of the network giving room to multiple steady states. The condition was formulated as a (non convex and multimodal) optimization problem, to be solved by global optimization algorithms. Before the search, it was needed to partition the parameter space in regions with different qualitative behaviour. The parameter space was then characterized depending on whether the optimization algorithm could find a solution or not, i.e., depending on whether or not we could find a point (or a set of discrete points) in the parameter space fulfilling the multistationarity condition.

In this work, we formalize the geometric intuition in [29] to derive a sufficient condition for multistationarity which is general, i.e. applies to networks where the dimension of the equilibrium manifold is lower, equal or greater than the deficiency. The general condition is stated in a formal context, and a proof of its validity in arbitrary dimensional spaces is provided. In addition, we present a method to search the condition through the parameter space with several advantages over global optimization methods. On the one hand, it is capable of finding the regions where multistationarity condition is fulfilled, without requiring the a priori partitioning of the parameter space in areas of different qualitative behavior. On the other hand, the method allows the characterization of the multistationarity regimes of a biochemical reaction network in a reliable manner, i.e. those regions in the parameter-state space leading to multiple steady states are ensured to be enclosed by the solution set.

### Fundamentals

Following CRNT classical description (see Feinberg’s Lecture 3 in [30] for details), we consider a generic reaction network involving 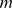 species 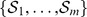 participating on a given set of irreversible reaction steps. Their concentrations 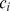 are collected on a vector 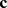 defined on the space 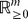 we will refer to as *the species space*. In the following, we write 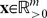 if 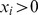 for all 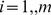 and 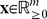 if 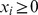 for all 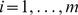. Each reaction step will be represented by an arrow which connects two particular combination of species, thus indicating how a given set of reactants is transformed into a certain set of products. The set of species at both extremes of the arrows are known in CRNT as *complexes*. The set of all complexes connected by reaction steps conforms the reaction network which is represented by a directed graph (C-graph or graph of complexes), where arrows (edges) indicate the reaction steps and the nodes correspond with the complexes (see Figure 2 for an example).

Let 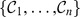 be the set of complexes of the network. To each complex 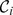 we associate a set 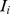 of integer elements which collects the indexes of those complexes that are directly reached from 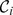 and a pair of vectors 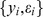. The set 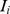 can be formally defined as:
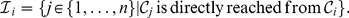



Vector 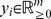 contains the molecularities of the species in complex 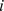, and 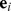 is a vector of the standard basis of 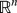 such that for every 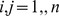:
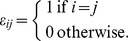



The complete set of edges in the graph is constructed by connecting 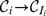 for all complexes 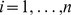. Every edge in the graph (or reaction step) directly linking complex 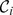 to complex 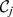, has its corresponding reaction rate, of the form:
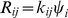
(1)where 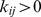 is a constant parameter and 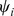 a scalar function:



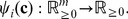
(2)In what follows we assume that the reaction rates are mass action. Thus each function 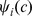 takes the form:
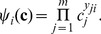
(3)


Provided that 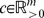 (i.e. it is a strictly positive vector), the expression (3) can also be written as:
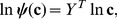
(4)where the natural logarithm operator 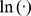 acts on any vector element-wise.

The C-graph of a reaction network is composed by a number 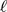 of “isolated” sub-graphs known in CRNT as *linkage classes* 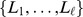, (see Lecture 3, page 14 in [30] for a complete discussion), each containing a number of complexes 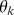 so that:
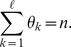



The reaction network presented in Figure 2 consists of two linkage classes: one involving complexes 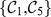, and the other involving complexes 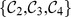. Each linkage class 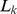 is accompanied by a vector 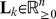 of the form:
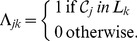
(5)


Complexes within a linkage class are linked by sequences of reaction steps (or equivalently by sequences of arrows) defining paths. Two complexes are strongly linked if they can be reached from each other by directed paths (every complex is strongly linked to itself). The maximal set of strongly linked complexes is a strong terminal linkage class if no other complex can be reached from any of its elements. A linkage class that is also a strong terminal linkage class is said to be *weakly reversible*. Weakly reversible networks are those composed of weakly reversible linkage classes. The network depicted in Figure 2 is an example of a weakly reversible network.

#### The dynamics of chemical reaction networks

Making use of the above definitions, the time evolution of species concentrations can be described by a set of ordinary differential equations of the form
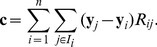
(6)


This system can be rearranged into the more familiar form, extensively employed in the context of CRNT:
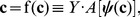
(7)where vector 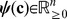 contains as entries the scalar functions 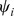 (2). In what follows, we will refer to the space 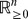 in which every 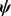 is defined as *the space of complexes*. The mapping 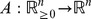 is such that:
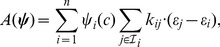
(8)and 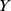 in (7) is the 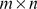 molecularity matrix, with columns being the molecularity vectors 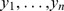 of complexes 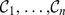. The right hand side of equation (7) 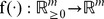 is known in CRNT as the species formation function [18].

Trajectories of system (7) are constrained in the concentration space by some invariants of motion to lie on convex regions within the non-negative orthant known as reaction polyhedrons [31], or *stoichiometric compatibility classes* in the CRNT formalism (see Lecture 2, page 17, Definition 2.9 in [30]).

In order to characterize these regions, and suggested by the structure of the operator 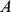 (Eq. (8)), let us first consider the subspace spanned by vectors 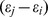, where the image of the operator 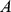 lies. The subspace is formally defined as:
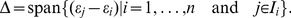
(9)


It must be noted that the number of independent vectors 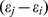 per linkage class is 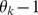, and vectors from different linkage classes are independent, thus the dimension of Δ is 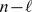. Related to Δ there is another subspace we define as follows:
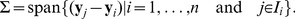
(10)


The subspace 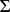 is known in CRNT as the *stoichiometric subspace*, where the species formation function 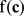 lies, since from (7) and (8) we also have that:
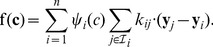
(11)


Let 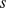 be the dimension of 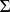 and 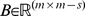 a full rank matrix that spans column-wise the orthogonal complement 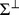.

A linear vector-valued function of the form 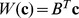 characterizes the invariants of motion, since it is constant along the trajectories defined by (7) for any initial condition 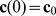. This can be shown by differentiating 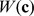 along (7) so that:
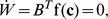
where the last equality follows because by construction 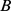 is orthogonal to the stoichiometric subspace 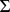. Integrating this expression in time and taking into account the initial condition 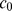 we get that:



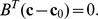



Each equation in the above expression is called a *conservation law* [32]. In this way, trajectories are constrained to regions that result from the intersection of the non-negative orthant 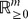 with any linear variety associated to the stoichiometric subspace. These regions we will refer to as reaction polyhedrons (equivalently stoichiometric compatibility classes), can be formally defined with respect to a reference concentration vector 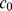 as:
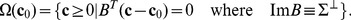
(12)


Mass conservation is a special case of invariance which leads to a particular class of polyhedrons, namely those which intersect every axis in the concentration space (in this case every species participate in at least one conservation law). Other polyhedrons are possible being parallel to some axis as it is the case of the example. In the extreme case (no species participating in conservation laws) the stoichiometric subspace spans the whole concentration space so that the orthogonal complement is the zero vector (the only column of *B*). In this case the reaction polyhedron is 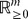.

#### The nature of equilibrium points

Next we summarize some results from CRNT to be used in the contribution concerning possible equilibrium solutions of (7), namely vectors 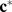 such that 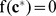. In what follows, we will concentrate on *weakly reversible* networks which in addition, for any initial condition 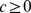 produce equilibrium points in the interior of the positive orthant. Note that if the trajectories lie in the interior of the positive orthant the networks under study are *persistent* [32] (i.e. those which for any initial condition 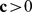 produce trajectories lying in 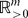).

Any equilibrium point 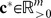 for (7) will satisfy either:
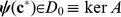
(13)or



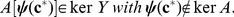
(14)Related to condition (14) there exists a subspace 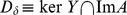, we will refer to as the *deficiency subspace*, which plays a central role in CRNT. Its dimension is called the deficiency 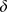 and can be computed by making use of the standard relationship between the dimensions of the domain, kernel and image of a linear transformation (see [30], Lecture 4, page 23 in the proof of Proposition 4.7). For weakly reversible networks, the terms in the relationship coincide with the dimensions of subspaces Δ (see (9)), 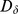 and 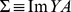, respectively. The final expression then reads:
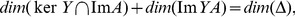
(15)since from previous discussion 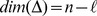 and 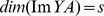, we finally get for 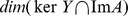:



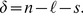
(16)It must be noted that as stated in the so-called *deficiency zero theorem* [30] (Theorem 5.1 in Lecture 5, page 2), any equilibrium point fulfilling (13) will be stable and unique in each compatibility class. Consequently, multistationarity, that is to say multiple equilibria, can only occur for 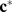 satisfying (14). For this reason, any weakly reversible reaction network of zero deficiency possesses a unique and stable equilibrium point (associated to 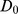) per stoichiometric compatibility class. This result remains valid independently of the values taken by the reaction rate constants.

In a previous paper [29], we have exploited the graph structure of biochemical networks to obtain an expression of the locus of equilibria –the set of points 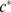 such that 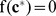 in (7)– in terms of as many parameters as the deficiency of the network. For a class of networks (the so called *proper networks*) we were able to partition (by continuation of variation parameters associated with the deficiency) the space of kinetic parameters in regions with different qualitative dynamic behavior. Exploiting the concept of equilibrium as a intersection between solutions satisfying (14) with the so called reaction polyhedron [31], conditions on the parameters of the network leading to multiple steady states were found for networks where the manifold dimension and deficiency coincide.

Here we start from this insight to provide a general condition for the existence of multistationarity, valid for weakly reversible mass action networks.

## Analysis

### The Locus of Equilibria

We present in this section a canonical expression for the locus of equilibria, that is to say the set of all possible feasible equilibrium solutions in terms of the kinetic parameters of the network. In what follows, we will refer to this locus as the *equilibrium manifold*. Mathematically it corresponds with an algebraic variety which results from the intersection of two other varieties: the *family of solutions* and the *mass action manifold* to be described below.

In preparing for the description and for simplicity, we assume that the molecularity matrix *Y* is full rank and 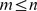. Furthermore, we assume that the *m* independent molecularity vectors 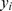 are distributed among linkage classes so that each linkage has at least one independent vector. For such networks, and without loss of generality, let us number the first 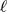 complexes so that each belongs to a different linkage class (note that by previous assumption 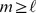), and so that the first 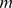 columns of the matrix 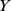 are linearly independent.

**The family of solutions** is a linear variety 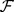 defined in the space of complexes by:
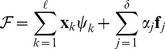
(17)where 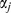 are given real numbers, and vectors 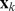, 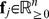 are solutions of the following equations:
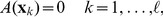
(18)

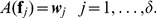
(19)


The set of vectors 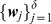 that appears in (19), defines a basis for the deficiency subspace 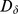. As proved in [29], the elements of a basis for 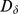 can be obtained from the left kernel of the matrix:
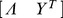
(20)where *Y* is the molecularity matrix and 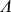 is the 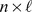 matrix with columns being the vectors 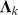 defined in (5).

Vectors 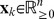 for 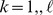 constitute a basis for the kernel of 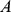 (Eq. (8)). As stated in Proposition 4.1 of [30] (Lecture 4, page 10), for weakly reversible networks the dimension of the kernel of 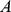, and therefore the number of vectors of the basis, coincides with the number of linkage classes. Actually, the same holds for networks other than weakly reversible, provided that they have one terminal linkage class per linkage class. The same Proposition prescribes for each element of the basis a nonnegative vector. In particular, each vector 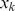 associated to a linkage class 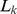, will have entries of the form:
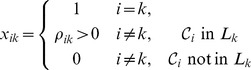
(21)where parameters 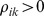 are functions of the reaction constants within the linkage class 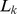. The functional relation will be formally represented as 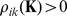 where 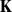 is the vector of kinetic rate constants. A proof of Proposition 4.1 is out of the scope of this contribution. It cannot be found in [30] either. However, two alternative proofs can be found in the literature: one based on Perron-Frobenius theorems applicable to weakly reversible networks [33]. The other more graph theoretically oriented has been proposed in [34].

Similarly, any entry 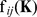 of vectors 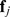 is a function of kinetic parameters within the linkage class containing the complex 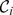. Explicit expressions relating parameters 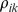 and 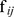 (for 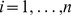, 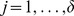) with the original kinetic constants are obtained by solving Eqs. (18) and (19).

It must be noted that by construction, 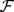 in Eq. (17) under linear transformation *A* produces vectors:
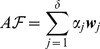
(22)which correspond with elements of the deficiency subspace 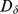. For the particular case of 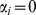 for every 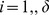, 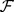 characterizes elements of 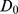 (i.e. the kernel of 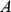). In this way, Eq. (17) provides a complete parametrization of complexes 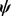, leading to equilibrium solutions.

**The mass action manifold** is a nonlinear algebraic variety 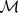 defined in the space of complexes by:
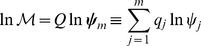
(23)where matrix 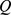 is of the form:



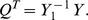
with 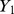 being a matrix containing the first 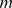 columns of the molecularity matrix 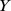. Note that each element 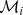 can be written as:



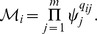



Expression (23) is the equivalent of (4), but defined in the space of complexes instead of the concentration space. The relationship between both spaces is given by the bijective mapping:
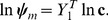
(24)


**The equilibrium manifold** is the algebraic variety which results from the intersection in the space of complexes of the family of solutions 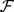 and the mass action manifold 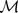. Formally the intersection can be expressed as:
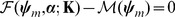
(25)where 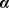 is the vector that collects all the 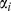 parameters for 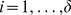. For a given parameter vector 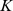, the equilibrium manifold can be written as:
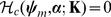
(26)where



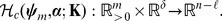
(27)On occasions it may be convenient to transform the algebraic variety by means of (24) to its equivalent in the concentration space, namely:
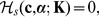
(28)where:



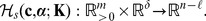
(29)It should be noted that given a rate constant vector 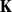, function 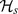 (or equivalently 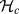) is continuous and differentiable. In addition the dimension of the expression (28) (or equivalently (26)), either in the space of complexes or in the species space is 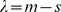. This is so since 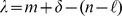, and by (16) 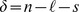.

### Condition for Multistationarity

This section contains the main result of the contribution namely a condition for a given network to have multiple (positive) equilibria within the same stoichiometric compatibility class. The condition is geometric in essence and makes use of the equilibrium manifold, expressed either in the space of complexes (26) or in the species space (28), and the linear variety that defines the set of reaction polyhedrons (12).

**Figure 1:**
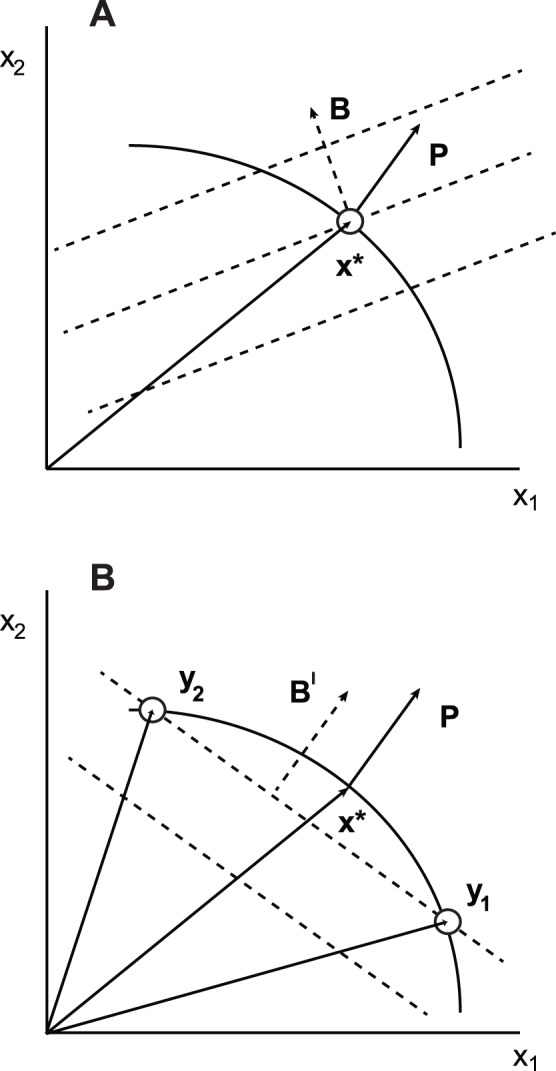
Schematic representation of the geometric condition. Solid and dashed lines represent nonlinear and linear varieties, respectively in the two dimensional 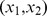 space. Vector 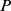 is perpendicular to the curve (nonlinear variety) at 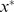. 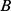 and 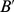 in Figure (a) and (b) respectively, denote vectors perpendicular to the linear variety (straight lines defining compatibility classes). In Figure (a) vector independency leads to one intersection per compatibility class, while in Figure (b) vector alignment leads to two different intersections (indicated by 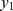 and 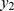).

The underlying idea behind the condition relates to the question of whether or not a given set of equations can accept more than one solution. A formal statement and discussion of this question is presented in the Appendix S1 for a general class of functions defined in 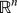. There we present the necessary mathematical background as well, but first let us illustrate the basic concept on a simple two dimensional case depicted in Figure 1.

The example consists of a nonlinear manifold (the continuous curve) and two possible families of linear varieties represented by dashed lines with gradient vectors 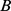 and 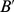 respectively. Vector 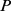, perpendicular to the curve at 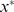, is the gradient of the curve at 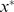. This vector also defines the tangent subspace to the curve at that point. As it can be observed in Figure 1 A, all linear varieties associated to vector 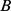 intersect the curve in just one point so no multiple solutions are expected.

On the other hand, some linear varieties associated to 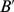 in Figure 1 B intersect the curve in two points 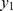 and 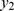 what corresponds with two different solutions. What differs between Figures 1 A and 1 B is the relative orientations of the curve and linear variety gradients. Thereby vector alignment (or linear dependency) is what seems to be at stake to determine the number of solutions. In this way, multiple solutions are expected to appear whenever vector alignment takes place. This is the notion we take advantage of and extend to higher dimensional manifolds (i.e. the equilibrium manifold).

As discussed in the Appendix S1, essentially all is needed is the equilibrium manifold to be locally smooth. If at a given point in the space of concentrations this is the case, hyperplanes secant to the equilibrium manifold can be constructed in a small neighborhood of the point by parallel translation of the corresponding hyperplanes tangent at that point (see Figure S1). Multiple solutions are then possible if the hyperplanes coincide with a given reaction polyhedron.

In the remaining of the section the results discussed in the Appendix S1 will be adapted to detect multiple equilibria associated to the equilibrium manifold. To that purpose a 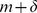 (equivalently 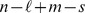) dimensional space will be employed, which includes the variables:
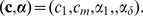



It must be pointed out that since the map (24) is bijective, the condition can be established either in the species space (*c* variables) or in the space of complexes (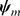 variables). However in the species space the reaction polyhedron is linear what simplifies the derivation of the condition. Thus for convenience, manifold (28) will be employed in first place. Nevertheless, some comments will be made at the end of the section on the condition expressed in the the space of complexes.

Firstly let us note that the Jacobian of 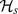 reads:
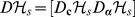
(30)where 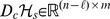 and 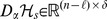 denote the Jacobians of 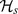 with respect to 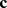 and 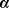, respectively.

In the space described by the variables 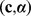 we express the linear variety associated to the reaction polyhedron (12) as:
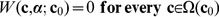
(31)where function 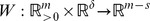 is of the form:



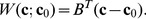
(32)Note that because 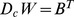 and 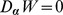 (i.e. a zero 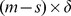 matrix), its Jacobian can be written as:
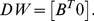
(33)

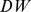
 is full rank by construction, since as discussed in the Fundamentals, 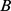 is a basis for the orthogonal complement of the stoichiometric subspace 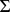.

For a given vector of rate constants 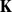, let 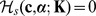 be continuous in the vicinity of a point 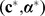. Note that by the implicit function theorem, this implies that 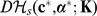 is full rank. Then we are under the conditions of Proposition A1 (see Appendix S1) where 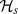 corresponds with 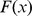, 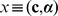 and 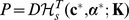. Furthermore, 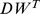 takes the place of *C* in matrix *G* in Corollaries A1 and A2. The corresponding 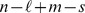 square *G* matrix then becomes:
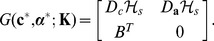
(34)We are now in the position to formally state the geometric condition.

**Proposition 1** Consider a reaction network with a given vector of rate constants 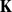, and let 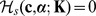 be continuous in its domain. If for any 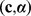 satisfying 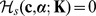 matrix 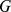 is full rank, the reaction network for 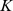 has at most one positive equilibrium solution per stoichiometric compatibility class.

**Proof:** the result follows directly from Corollary A1 (see Appendix S1), applied to the domain where the equilibrium manifold (29) is defined (i.e. positive concentration space).

**Proposition 2** Given a vector of rate constants 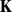, a sufficient condition for the reaction network to exhibit multiple (positive) steady states within the same stoichiometric compatibility class is that for at least some 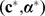 such that 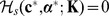, matrix 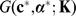 is rank deficient. Equivalently, a sufficient condition for the reaction network to exhibit multistationarity is that:
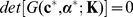
(35)
*for some 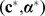.*

**Proof:** The result follows directly from Corollary A2 (see Appendix S1), applied to the domain where the equilibrium manifold (29) is defined (i.e. positive concentration space). Rank deficiency can be checked through expression (35).

The condition for multistationarity remains valid in the space of complexes, since the map (24) is bijective. In this space however, 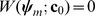 is nonlinear, although continuous for every 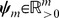 (namely in the interior of the space of complexes). This can be shown by using (24) to compute its Jacobian with respect to 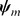, so that:
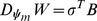
(36)with 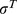 being of the form:
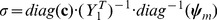
(37)where 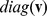 and 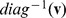 are the diagonal and inverse diagonal matrices operating over the vector 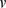, respectively.

The Jacobian 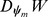 is full rank since 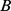 is full rank and 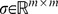 invertible. Continuity of 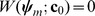 then follows from the implicit function theorem (see Appendix S1). In the space of complexes, Condition (35) from Proposition 2 should be checked on the matrix:
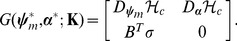
(38)


### Interval Based Search

In order to find the regions in the parameter space fulfilling the multistationarity condition we formulate a so called *continuous constraint satisfaction problem* (CSP) [35], [36] and solve it numerically by using interval methods [37]. Methods based on interval analysis allow mathematical operations to be carried out over real intervals instead of real numbers, and thus to represent a continuum of solutions to a given CSP by a finite number of intervals or boxes, the union of which encloses the solution set.

Let 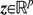 be the space where a set of constraints is defined. A domain in that space is constructed by interval variables 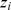 (for 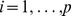) defined on closed real intervals 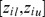, where subindexes 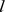 and 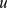 stand for lower and upper bounds, respectively. For 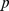 variables, the cartesian product of ◊interval domains 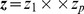 is called a box.

Following [36], a constraint satisfaction problem consists in finding an interval domain where a set of constraints 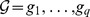 hold. This can be formally stated as:
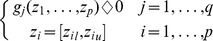
where constraints involve nonlinear analytic expressions and the symbol 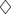 stands for either equality or inequality constraints, that is to say 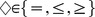. A solution of a CSP is an element of the search space which fulfills all the equalities and inequalities simultaneously.

In our case, the search space involves the elements of the rate constant vector 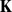 and the independent variables which characterize the equilibrium manifold (which equals 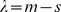, the manifold dimension). In this way, for 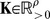, the number of variables is 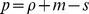.

Regarding constraints, the equality ones correspond with the equilibrium manifold plus condition (35) which adds up to 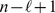 equations. On the other hand, inequality constraints must be imposed on the dependent variables that describe the equilibrium manifold, to ensure positivity of variables representing concentration and to search on non-zero 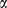 values.

Characterizing multistationarity regimes boils down to find the regions in the parameter-state space composed of the rate constants and independent variables which fulfill the constraints, i.e. computing all real feasible solutions of the corresponding CSP.

Solutions in interval methods are approximated by *subpavings* which consist of unions of boxes. Formally, a subpaving 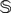 for a given region 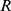 of the search space is defined as the union of nonoverlapping boxes approximating 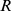. If we construct subpavings 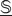 and 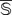 such that:
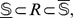
the region 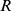 is bracketed between inner and outer approximations. The outer approximation is reliable [38], since it is guaranteed that the solution region is contained within 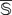.

Simple algorithms as SIVIA (Set Inverter Via Interval Analysis) proposed in [37] can be used to compute inner and outer subpavings by successive bisections and selections. Implementing interval algorithms requires environments supporting interval arithmetics such us the free available software package INTLAB, which provides an interactive environment within Matlab [39], [40]. Efficiency can be gained by branch-and-prune algorithms where a set of boxes that contains all the solutions of the CSP is computed, and then each box is reduced and split. The free software package REALPAVER [36] provides a modeling language and a generic branch and prune algorithm which combine different splitting strategies and pruning techniques.

### Example: Multistationary Regimes in an Autocatalytic Network

As a proof of concept, we make use of a simple autocatalytic network introduced by Edelstein [41], and already used as a case study for the bifurcation analysis in the context of biochemical systems [42]. Bistability has been shown for very simple autocatalytic systems like phosphorylation-dephosphorylation cycles with autocatalytic kinase [43].

**Figure 2:**
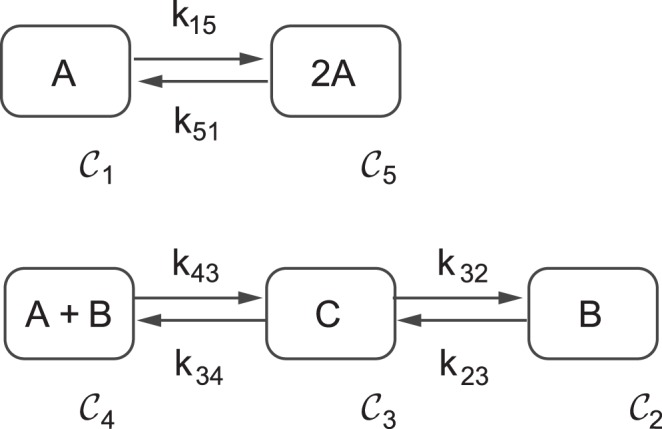
Graph of complexes (C-Graph) for the Edelstein network. The network involves three chemical species *A*, *B* and *C*. Nodes in the graph correspond with complexes, denoted by 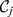 with 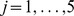. Arrows in the graph represent reaction steps. In this example every reaction is reversible.

The Edelstein network involves three species 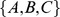, distributed into five complexes, 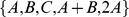. The C-graph for the Edelstein network is depicted in Figure 2, where the following index sets indicate complex interconnections: 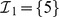, 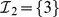, 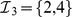, 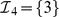, 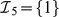. The reaction steps that correspond with the set of edges in the graph are presented in Table 1. The network has two different linkage classes: 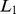 containing the complexes (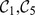), and 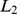 containing the complexes (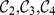). Their corresponding vectors 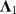 and 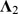 (see (5)) read:
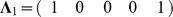


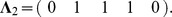
From the C-graph structure it follows that the network is weakly reversible since each linkage class is weakly reversible (in fact the network is reversible).

**Table 1:** Edelstein network reaction steps.

Reaction step	Rate
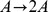	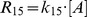
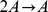	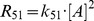
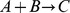	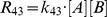
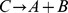	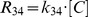
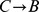	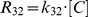
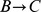	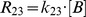

The species space is in this case three dimensional (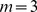) with concentrations 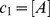, 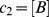 and 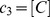. The molecularity matrix *Y* reads:
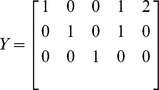
with rank equal to the number of species, i.e. 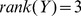. The dynamics of the reaction network can be encoded by Eq. (7), where the expression for 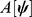 reads:



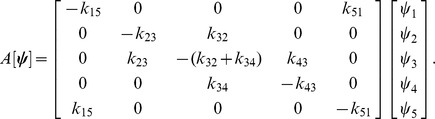



The elements of vector 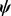 (mass action monomials) are related to concentrations by Eq. (3) so that:
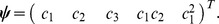



The time evolution of species concentrations results from substituting previous expressions for *Y*, 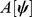 and 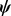 into Eq. (7) so that:
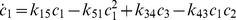


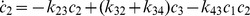


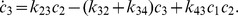



The stoichiometric subspace 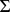 defined in (10) is in this case:
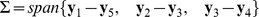
being its dimension 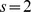 (i.e. two linearly independent vectors). The corresponding matrix *B* (with columns defining a basis of the orthogonal complement 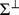) is:



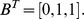



For reference concentrations 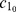, 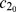, 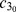 the linear variety (31) is a plane of the form:
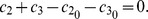



According to formula (16) we have that 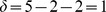. Thus the deficiency subspace 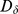 is one dimensional with the following basis computed from the left kernel of matrix (20):
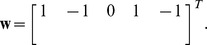



For this network, the expression for the family of solutions (17) takes the form:
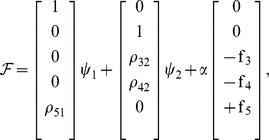
where the explicit expressions relating the parameters 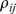 and 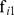 (subindex 1 is omitted), obtained by solving Eqs. (18) and (19), are given in Table 2.

**Table 2:** Edelstein network parameter equivalences.

Parameter	Equivalence
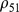	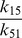
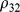	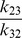
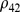	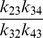
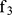	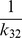
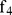	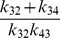
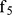	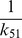

In order to compute the mass action manifold (23) we first select matrix 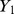 as that which collects the first 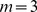 molecularity vectors:
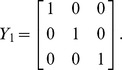



Matrix 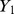 defines the bijective mapping (24). The resulting mass action manifold becomes:
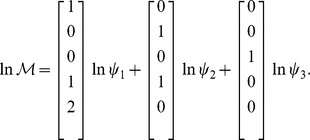



Using (25), we compute the equilibrium manifold as the intersection of the family of solutions with the mass action manifold. Its dimension is 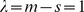 and its expression in the space of complexes (26) is given by the equations:
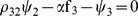


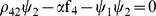


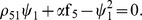



The bijective mapping (24) allows us to write the equations for the equilibrium manifold in the species space (28):
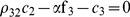
(39)

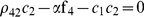
(40)

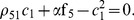
(41)


In order to derive the geometric condition defined by (35) in Proposition 2, we first compute the Jacobian of 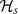 as described in (30), where:
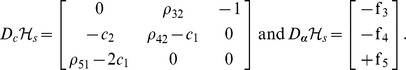



Matrix 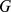 in (34) becomes:
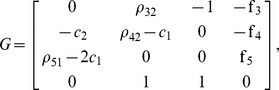
being its determinant:



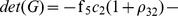


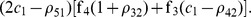



Setting 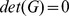 as in (35) and using the equivalences in Table 2, we get:
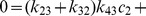


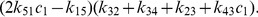
(42)


In the interior of the species space (i.e. that of positive concentrations), the first term at the right hand side of (42) is always positive, while the sign of the second term is conditioned by that of 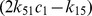. Therefore, the determinant can only vanish for values of 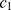 that make 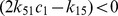. Equivalently, for 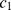 values within the open interval 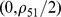, i.e. 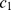 satisfying:
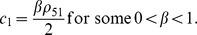



Substituting this expression for 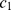 into (41) and its result in (40) we obtain, respectively:
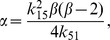
and



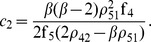



Since 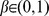 the term 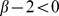. Thus for 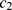 to be positive it is required that:
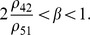



By substituting the expressions obtained for 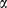 and 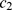 in the equilibrium manifold 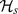, it can be deduced that 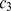 is also positive for every 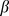 in the open interval 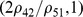. Summarizing, the network will show multiplicities for those parameters fulfilling (42) with:
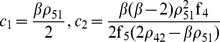
for 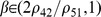. Note that multistationarity is ruled out for 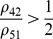 since 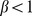.

Let us consider the set of kinetic constants given in Table 3. For 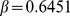 they satisfy the condition described by (42). The resulting values for 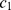 and 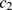 are:
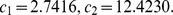



**Table 3:** Edelstein Network parameters.

Kinetic constant	Value
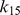	8.5
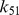	1
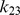	0.2
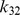	1
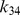	1
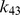	1

**Figure 3:**
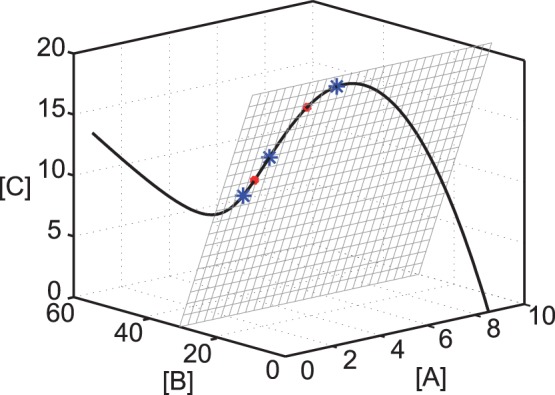
Equilibrium manifold for the Edelstein network. The corresponding parameter values are given in Table 3. Stars indicate steady states of the system for 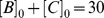. Dots indicate those points in the locus of equilibria where the rank deficiency condition is fulfilled.

**Figure 4:**
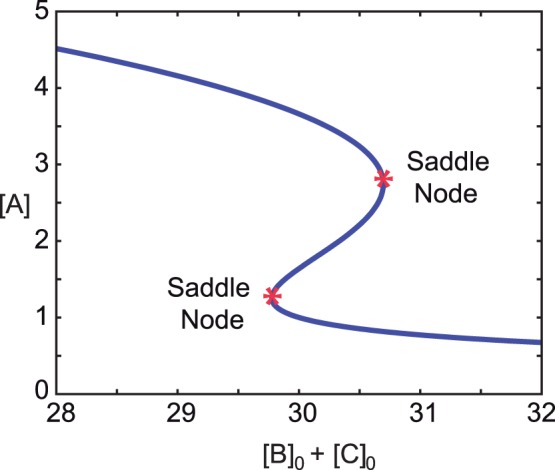
Equilibrium curve for the Edelstein network. The curve is obtained by varying 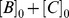, using the software Cl Matcont [46]. The kinetic parameters are kept fixed with the values shown in Table 3.

In Figure 3, the equilibrium manifold for these values of the kinetic constants is depicted, together with the reaction polyhedron corresponding to 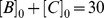. The manifold is one dimensional, and intersects the reaction polyhedron in three points, corresponding to three different equilibria. The points fulfilling the rank deficiency condition, corresponding to 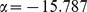 and 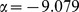 are also indicated. As it can be deduced from the figure, three steady states will exist for a range of the sum of initial concentrations 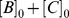. In fact, performing a continuation of the curve of equilibria by varying the values of 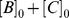 we obtain the curve shown in Figure 4, where two limit points or saddle node bifurcations appear for 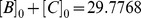 and 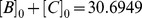. Within these values, corresponding to different positions of the reaction polyhedron, three steady states will exist. Note that the points fulfilling the rank deficiency condition indicated in Figure 3 correspond precisely with the bifurcation points in Figure 4.

Alternatively, interval methods can be employed to search for the condition given by (35). Here it is important to remark that the method allows searching for parameters and/or steady state values of species concentrations within multistationary regimes, provided some other parameter values and/or steady state concentrations fixed. To illustrate this, let us assume we are interested in the ranges of parameters allowing for multistationarity, and the steady state values of 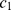, for a given steady state concentration of the species 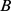 (i.e. 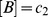). The variables in the constraint satisfaction problem are thus the free kinetic parameters and the steady state concentration of the species 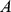.

**Figure 5:**
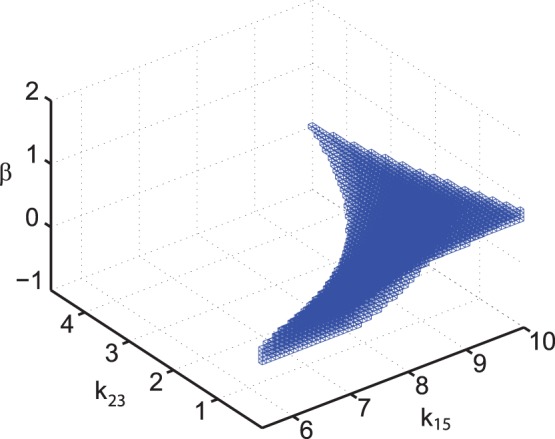
Outer approximation of the region of multistationarity for the Edelstein Network. The concentration 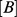 is fixed (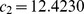), 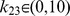, 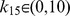, and values of 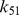, 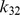, 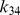 and 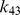 given in Table 3.

A three dimensional plot of the result is given in Figure 5 for 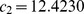, where the 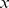 and 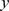 axis represent two of the kinetic parameters (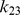 and 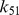), and the 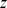 axis represents a function of the steady state concentration of the species 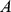 in the steady state. We represent 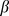 in the 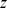 axis in order to facilitate the comparison with the results derived analytically. The corresponding values of 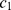 can be computed as 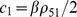.

## Discussion

In this work we present a method to compute the regimes of multiple steady states in biochemical reaction networks, i.e. the regions in the parameter space, or in the state-parameter space of network models leading to multistationarity.

The main result of the paper consists of a sufficient condition for multistationarity, demonstrated to be valid for weakly reversible networks of arbitrary dimension and deficiency.

The idea behind is based on the fact that one steady state is an intersection between the locus of equilibria, or equilibrium manifold, and the reaction simplex, or stoichiometric compatibility class. In search for the existence of multiple steady states in reaction networks, we explored the requirements for multiple intersections between these two varieties.

In a previous work of the authors [29] it was shown that, for networks with 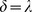, (or equivalently with 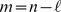), and fixed a kinetic rate vector **K**, the equilibrium manifold could be continued by the variation of the deficiency parameters of the network, and its qualitative behaviour evaluated through the derivative of the manifold with respect to the deficiency parameters. After partitioning of the parameter space in regions of different qualitative behaviour, global optimization algorithms [44] were used to check, within every region, whether multiple intersections between the manifold and the simplex were possible.

Here we use and extend this geometric insight to state a general condition of multistationarity for networks where the deficiency might be lower, equal or greater than the dimension of the equilibrium manifold. In this regard, [29] deals with a particular case of the general condition presented (and proved) here.

The evaluation of the multistationarity condition presented boils down to check the rank of a matrix which depends on the kinetic parameters, the concentrations, and the deficiency parameters. This matrix is systematically derived as indicated in the Analysis section, from the equations of the manifold and the mass conservation laws. In order to check the condition through the (state-)parameter space, we reformulate the problem as a constraint satisfaction one to be solved by interval methods.

For the purpose of characterizing the (state-)parameter space in terms of the capability for multiple steady states, methods based on interval arithmetics presents several advantages over classical global optimization methods [44]. On the one hand, they allow identifying regions, and not sets of discrete points, in the (state-)parameter space. In this way, there is no need to partition a priori the (state-)parameter space in regions with different qualitative behaviour. On the other hand, they ensure reliability of the solution [38], i.e. they guarantee that all the multistationarity regimes are enclosed by the solution regions.

As it has been commented in the Introduction, the CRNT provides particularly strong results to rule out multiple steady states based on the network structure irrespective of the network parameters. In this regard, this paper extends the results of the CRNT by introducing the kinetic parameters into the picture, giving a general condition for the appearance of multiple steady states.

In terms of applicability and performance, the method presented here is valid independently of the value of the network deficiency and dimension. The efficiency of the search will depend on the computational cost of the algorithm which increases in high dimensional spaces. In case we are only interested in finding points in the state-parameter space leading to multistationarity (for example if the goal is to decide only whether the network can exhibit multiple steady states or not), global optimization methods [44], [45] would perform much faster.

Once the condition for multistationarity is derived, and depending on the particular scenario we want to explore, the search can be performed considering some of the parameters and/or states to be fixed. In this way we obtain bifurcation diagrams in the desired low dimensional projections of the state-parameter spaces much more efficiently than using standard continuation techniques. For example, if we are interested in computing all the parameter sets giving multiple steady states for a particular value of the equilibrium concentration of one the species, we can perform the search keeping this concentration fixed.

To end up, we would like to stress the applicability of our method to standard problems appearing in systems and synthetic biology. The method can be particularly convenient, for example, to evaluate the robustness of the switching property for a given network. As remarked by [13], building a synthetic switch does not only require to start from a network structure allowing bistability, but such bistability must be sufficiently robust, meaning that the range of parameters leading to bistability is wide enough to enable a successful practical implementation. In this context, our method will efficiently characterize the regions of interest within the state-parameter space.

## Supporting Information

Figure S1
**Representation of the tangent and secant hyperplanes associated to a nonlinear manifold, in this case a curve, at 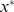; 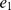 and 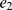 are unit vectors centered at 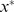; 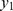 and 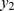 are two different points of the manifold.**
(EPS)

Appendix S1 (PDF)
