# Correction: Characterizing Multistationarity Regimes in Biochemical Reaction Networks

**DOI:** 10.1371/annotation/b5283cc5-5a93-4c96-abc4-c5112266fabe

**Published:** 2013-01-15

**Authors:** Irene Otero-Muras, Julio R. Banga, Antonio A. Alonso

There were errors in the Funding section. The correct funding information is as follows: This work was partially supported by the Spanish MICINN project MultiScales (DPI2011-28112-C04-03), the CSIC intramural project BioREDES (PIE-201170E018) and European FP7 project KBBE-2007-2-3-01 CAFE . One of the authors thanks partial support provided by the ”Salvador de Madariaga” Mobility Programme(2011-2012). The funders had no role in study design, data collection and analysis, decision to publish, or preparation of the manuscript.

