# Supplementary material for: Characterizing Multistationarity Regimes in Biochemical Reaction Networks
Source: PLoS One. 2012 Jul 3;7(7):e39194. doi: 10.1371/journal.pone.0039194 (PMC3389020; doi:10.1371/journal.pone.0039194)
Supplement: Appendix S1 — (PDF) [file pone.0039194.s002.pdf]

Appendix S1

Here we state and discuss in a formal context the arguments used in support of the geometric condition and its application for detection of multiplicities. In its most general form, the problem to be addressed can be stated as follows:

Consider the functions $F : \mathbb{R}^n \to \mathbb{R}^m$ and $W : \mathbb{R}^n \to \mathbb{R}^{n-m}$ (n and m, with $m < n$, denote here the dimensions of arbitrary spaces). Let $W(x)$ be linear so that $W(x; x_0) = 0$ describes a linear variety defined by $n - m$ hyperplanes passing by some $x_0 \in \mathbb{R}^n$.

Let $C = D_x W^T(x; x_0)$, where $D_x W$ denotes the Jacobian of W with respect to x, be the matrix that has as columns a basis of the orthogonal complement to the subspace that induces the linear variety. The question is whether or not the system:

$$\begin{aligned} F(x) &= 0 \\ W(x; x_0) &= 0 \end{aligned} \tag{1}$$

can have more than one solution for some $x_0 \in \mathbb{R}^n$.

In order to discuss the main elements of the approach let us partition the space $\mathbb{R}^n$ in two subspaces $\mathbb{R}^m$ and $\mathbb{R}^{n-m}$, and assume that the function $F(u, v) : \mathbb{R}^m \times \mathbb{R}^{(n-m)} \to \mathbb{R}^m$ is analytic (i.e. continuous and differentiable up to any order). Denote by $D_u F$ and $D_v F$ the Jacobians of F with respect to vector $u \in \mathbb{R}^m$ and $v \in \mathbb{R}^{n-m}$, respectively.

We say that $F(u, v) = 0$ is continuous in some vicinity of $x^* = (u^*, v^*) \in \mathbb{R}^n$ if it satisfies the implicit function theorem [1], which reads as follows:

Suppose $F(x^*) = 0$ for some $x^* \in \mathbb{R}^n$, and that the matrix $D_u F(x^*)$ is nonsingular. Then there exist some open sets $U \subset \mathbb{R}^m$ and $V \subset \mathbb{R}^{n-m}$ containing u^* and v^* respectively, and a continuous function $H : V \to U$ such that $u^* = H(v^*)$ and $F(H(v), v) = 0$ for all $v \in V$. Moreover $H(v)$ is uniquely defined.

Let e_1 and e_2 be two unit vectors as in Figure S1, and select a small distance h around x^* so that the vectors:

$$y_1 = x^* + (1/2)he_1 \tag{2}$$
$$y_2 = x^* + (1/2)he_2 \tag{3}$$

belong to the open set $U \times V$. By the mean value theorem [1], for each continuous and differentiable function $F_i(x)$ with $i = 1, \ldots, m$, there exist some number $t_i \in (0, 1)$ and a vector $z_i = (1 - t_i)y_1 + t_i y_2$ such that:

$$F_i(y_2) - F_i(y_1) = D_x F_i(z_i)(y_2 - y_1). \tag{4}$$

Choosing h sufficiently small, we have that $D_x F_i(z_i) \to D_x F_i(x^*)$, so this way $F_i(y_2)$ can be reasonably approximated [1] as:

$$F_i(y_2) \simeq F_i(y_1) + D_x F_i(x^*)(y_2 - y_1). \tag{5}$$

Repeating this argument for all the functions $i = 1, \ldots, m$ we have:

$$F(y_2) \simeq F(y_1) + D_x F(x^*)(y_2 - y_1). \tag{6}$$

With these preliminaries we are in the position to state the following proposition:

Proposition A1. *Let $F(x) : \mathbb{R}^n \to \mathbb{R}^m$ be analytic, and $F(x) = 0$ continuous in some vicinity of x^*. Let y_1 be a solution sufficiently close to x^*, there exists at least some $y \neq y_1$ which simultaneously solves:*

$$\begin{aligned} F(y) &= 0 \\ P^T(y - y_1) &= 0 \end{aligned} \tag{7}$$

where $P = D_x F^T(x^*)$.

Proof: Since $F(x) = 0$ is continuous around x^* it satisfies the conditions of the implicit function theorem. Thus two vectors y_1 and y exist close enough to x^* so that $F(y_1) = F(y) = 0$ (i.e. the vectors are in the $U \times V$ open set as discussed above). Also, since (6) applies, we have that $D_x F(x^*)(y - y_1) = 0$ which coincides with (7). $\square$

Remark *Note that the columns of P are linearly independent, at least in the neighborhood of x^* which is where the implicit function theorem holds.* $\triangle$

What Proposition A1 presents is a way to construct hyperplanes secant to a manifold in the neighborhood of a given regular point. Here are two direct consequences to be drawn from the discussion and Proposition A1:

Corollary A1. *If the $n \times n$ matrix $G = [\ P \quad C\ ]^T$ is full rank, then problem (1) has at most one solution for any x_0, at least in the vicinity of x^*.*

Proof: Let y_1 be a solution of (1) in the vicinity of x^*, as described in Proposition A1. Any other solution, at least in the vicinity must be of the form $y = y_1 + \tau$ where the vector τ must simultaneously satisfy $P^T \cdot \tau = 0$ and $C^T \cdot \tau = 0$. Since G is full rank, $\tau = 0$ so the solution is unique. $\square$

Corollary A2. *If the $n \times n$ matrix $G = [\ P \quad C\ ]^T$ is rank deficient, then problem (1) has more than one solution in the vicinity of x^*, at least for some x_0.*

Proof: Since G is rank deficient, a nonzero vector τ exists simultaneously satisfying $P^T \cdot \tau = 0$ and $C^T \cdot \tau = 0$. Let y_1 be one solution of (1) in the vicinity of x^* as described in Proposition A1, then $y = y_1 + \tau$ is another solution of (1) with $x_0 = y_1$. $\square$

References

[1] Nocedal J, Wright JS (2006) Numerical Optimization. Springer, London.
